# Re-Evaluation of Vascular Histogenesis in the Root Tips of Selected Species in the Poaceae Using New Methods: Analysis of the Plerome, Vascular Initials, Pericycle and Late-Maturing Metaxylem Vessels

**DOI:** 10.3390/plants13060910

**Published:** 2024-03-21

**Authors:** Yasushi Miki, Susumu Saito, Teruo Niki, Daniel K. Gladish

**Affiliations:** 1 Image Processing Section, MikiOn LLC, 593-1-102 Kunugida, Tokyo 193-0942, Japan; yas@mikion.tokyo (Y.M.); mdjmk210@ybb.ne.jp (S.S.); teruo-niki@hb.tp1.jp (T.N.); 2 Department of Biological Sciences, Miami University, 1601 University Blvd., Hamilton, OH 45011, USA

**Keywords:** 3D image processing, root apical meristem (RAM), vascular initials, late-maturing metaxylem vessel (LMX), pericycle, plerome, barley, maize, rice, teosinte

## Abstract

Serial sectioning and 3D image reconstruction methods were applied to elucidate the structures of the apices of root vascular cylinders (VCs) in taxa of the Poaceae: *Zea mays* “Honey Bantam”, *Z. mays* ssp. *mexicana*, *Hordeum vulgare* and *Oryza sativa*. The primary and nodal roots were investigated. Observations were performed using high-quality sectioning and 3D image-processing techniques improved and developed by the authors. We found that a quiescent uniseriate plerome was located at the most distal part of each VC. Vascular initials were located immediately basipetally to the plerome as a specific uniseriate layer that could be classified into central and peripheral initials that produced all the cells in the VC. No supplying of cells from the plerome to the vascular initials was observed. Numerical analysis revealed a “boundary point” along the root axis where the rate of increase of the vascular cell number markedly declined, and the VC diameter, number of vascular cells, and number of late-maturing metaxylem vessels (LMXs) at that point showed a similar relationship among the taxa and the types of roots examined (primary vs. nodal). The plerome and vascular initials layer can be considered independent after seed germination in these taxa. A boundary point at which procambial cell proliferation sharply declined was identified. The diameters of the VCs, number of LMXs, and number of vascular cells at the boundary point were found to be strongly related to each other.

## 1. Introduction

Historically, plant anatomists have had a strong interest in learning about the structure and histogenesis of plant root apical meristems (RAMs). In his pioneering work on embryogenesis in monocotyledons and dicotyledons (notably, species of *Capsella* and *Allium*, among others), von Hanstein [1] described the idea of dividing the promeristems of roots and shoots into three distinct groups of cells that persisted, called histogens (histogen theory): the dermatogen, which produces dermal tissue (rhizodermis/epidermis, respectively), the periblem, which produces the ground tissue of the cortex, and the plerome, which produces the central vascular cylinder (VC), in the case of roots. This early proposal eventually faced competition from alternative interpretations of anatomical data, notably the Körper–Kappe theory of Schüepp [2] (described by Clowes, [3] and comprehensively reviewed by Heimsch and Seago, [4]). Clowes [3] described Hanstein’s interpretations [1] as “unrealistic” because they “fail to account properly for the organization observed”. He reported that Schüepp’s interpretation [2] described a “cytogenetic centre” that produced a Körper (body) that gives rise to inner tissues, such as the vascular tissue, and a Kappe (cap), which would give rise to the ground tissue of the cortex and epidermis. Clowes [3] specified that neither the Körper nor the Kappe was a histogen. Later, Clowes [5] described what came to be called “the Quiescent Center”, a region in the promeristem of roots with little or no cell cycling activity.

In more recent times, von Hanstein’s idea [1] has largely only been associated with young roots of species described as being of the strictly “closed type”, wherein the major root primary tissue systems (dermal, ground, vascular, and root cap) each ultimately derive from distinct tiers of histogen cells located transversely within the promeristem [4,6,7]. It is known, however, that the closed organization (below) may become more “open” over time in some species [4]. Therefore, the developmental patterns specific to the plerome that von Hanstein (1), and later von Guttenberg (6), suggested are variable. In particular, the plerome as initials for the entire VC has been contested in *Zea mays* [8].

In a report on wheat (*Triticum*) and corn (*Zea*), Clowes [9] proposed a new concept for the structure of root apices. He stated that initials occupy a relatively large area at the apex of the VC, but he did not specifically mention the relationship between the plerome and initials. Von Guttenberg [6] reviewed the tissue anatomy at the root apex of various angiosperms and disputed the quiescent center concept. Though he classified the gross structures of root apices into two types, the “closed type” and “open type”, the characteristics and definitions of the plerome and actual initials were not fully explained. In particular, he did not clearly distinguish the placement of the initials with respect to the plerome and their relevant contributions to VC development.

Heimsch and Seago [4] investigated and reviewed the organization of the various RAMs in angiosperms. They classified several patterns of the cortical and rhizodermal initials of root apices, but they did not specifically describe the development of the VC structure, especially with respect to the role of the plerome and the actual initials that ultimately result in the production of the various VC tissues (xylem, phloem, pericycle, and, in some species, parenchymatous pith). Over the past several decades, researchers have studied tissue pattern-making via the gene expression patterns of mutants of *Arabidopsis thaliana*, e.g., Dolan et al. [10]. Baum et al. [7] revealed the developmental process of the reorganization of the early cortex and vascular cylinder of the RAM of *A. thaliana* over time.

Only a few details were known about the histogenesis of the procambium in monocot plant roots because of not having sufficiently useful tools to elucidate the problem. Heimsch [11] and Kawata et al. [12,13,14] carefully observed the structure of the root apex of barley (*Hordeum sativum*) and rice (*Oryza sativa*), respectively, but they did not describe details of the structure or function of the plerome and associated initials. Saito et al. [8] reported the relationships among the initials and late-maturing metaxylem vessels (LMXs) in the VCs of *Zea mays* ssp. *mexicana* and *Z. mays* “Honey Bantam”. However, they could not clarify all the cell-to-cell relationships and meristematic functions of the plerome, relevant initials, and the LMXs using their improved traditional methods.

Here, we also contemplate unasked questions about species in the Poaceae with respect to whether primary roots (emerged radicles) have the same apical structure as nodal roots (adventitious roots) produced by stems. There have been few reports that have tried to compare the apical structures of primary versus nodal roots in the Poaceae [15].

High-quality section processing [16] and three-dimensional (3D) image reconstruction from serial sections [17,18] were effective methods for clarifying the spatial configurations of cell and tissue features. These methods allowed our current analysis of the cell file origins and histogenesis in the vascular tissue of root apices.

The present report elucidates the structure and development of the plerome cells, pericycle, vascular initials, and LMXs in the procambium of four poaceans, and it provides a numerical characterization of these activities. Furthermore, it describes the differences in the tissue structure between primary and nodal roots.

**Figure 1:**
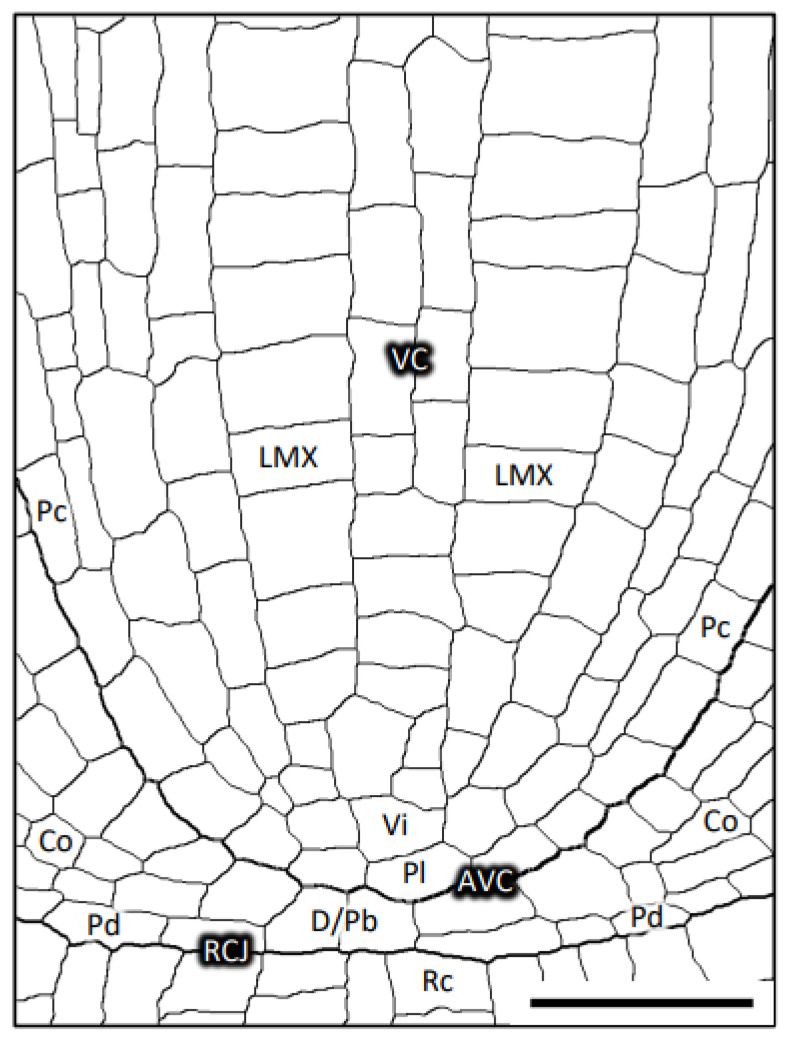
Diagram of a longitudinal median section of the teosinte primary root. AVC, the outer margin of the pericycle and plerome at the apical end of the VC; Co, cortex; D/Pb, dermatogen–periblem complex; LMX, late metaxylem vessel cell file; Pc, pericycle cell file; Pd, protoderm; Pl, plerome; Rc, root cap cell; RCJ, root cap junction; Vi, vascular initials layer; VC, vascular cylinder. Scale bar = 50 µm.

For the relevant terminology in this report, a diagram of a median longitudinal section of a primary root of teosinte is shown for reference as a typical example of the root apical meristem of the taxa we evaluated in the Poaceae (Figure 1).

## 2. Materials and Methods

### 2.1. Plant Materials

Caryopses (dry fruits commonly known as grains) of sweet corn (*Zea mays* “Honey Bantam”) and teosinte (*Z. mays* ssp. *mexicana*) from Snow Brand Seed Co., Ltd., Sapporo, Japan; of barley (*Hordeum vulgare*) from Dr. Yoshiaki Inukai, International Center for Research and Education in Agriculture, Nagoya University, Nagoya, Japan; and of rice (*Oryza sativa* “Hitomebore”) from Noguchi Seed Co., Ltd., Saitama, Japan, were used.

For the primary roots (radicle/tap root), the cultivation methods were modified after Gladish and Niki [19]. The grains were surface sterilized for 5 min in 10% (*v*/*v*) household chlorine bleach with 10 drops per liter of Tween 20 (Sigma-Aldrich, Burlington, MA, USA), then rinsed in sterile distilled water. Twenty grains each were sown in 1 L beakers containing autoclave-sterilized, moistened vermiculite and then incubated at a constant 20 °C in a continuously dark growth chamber for 4–5 d.

For the nodal roots, the grains were sown in fields at Hachioji, Tokyo, Japan, (35°40′ N 139°19′ E) from April to August. The daytime temperature during the period ranged from 20 to 30 °C. The nodal roots were collected approximately 1 mo after planting during the above period and were ca. 10 cm in length.

### 2.2. Preparation for Light Microscopy (LM)

The procedures used for LM were modified from Niki et al. [20]. Root tip segments of the primary and nodal roots were taken from the selected roots (1.5–2.5 cm and 3.0–8.0 cm long, respectively), immediately fixed in 4% (*w*/*v*) paraformaldehyde in 0.1 M phosphate buffer, and gently shaken overnight at room temperature. Following fixation, the specimens were rinsed in the buffer, dehydrated in an ethanol series, embedded in Technovit 7100 resin (Heraeus Kulzer GmbH, Wehrheim, Germany), and transverse and longitudinal serial sections were cut with a thickness of 1 and 2 μm, respectively, on a Reichert-Nissei UCT ultramicrotome (Leica Ltd., Tokyo, Japan).

The RNase-treated sections were prepared to create high-resolution and high-contrast LM photomicrographs according to Niki et al. [17] as follows. The sections mounted on glass slides were treated with ribonuclease A (RNase A; Sigma Chemical, St. Louis, MO, USA) at 60 μg/300 μL 0.05 M phosphate buffer solution (pH 7.2), which removed RNAs that would otherwise cause heavy staining because of their cytosolic abundance [21] and obscure other details in the section image. The slides were kept in an incubation box at 100% humidity and 37 °C for 2–3 h. After washing with distilled water (DW), these sections were stained with 0.1% (*w*/*v*) toluidine blue O (TB) (Electron Microscopy Sciences, Hatfield, PA, USA) at 45 °C for 4–5 min and rinsed with distilled water.

The sections were observed with a Leica DMLB light microscope (Leica Microsystems GmbH, Wetzlar, Germany) equipped with TU Plan Fluor objective lenses (20× ∞/0 or 50× ∞/0, Nikon Corp., Tokyo, Japan) and photographed with a Canon EOS 5D Mark II digital camera (Canon Inc., Tokyo, Japan), according to Niki et al. [16].

### 2.3. Image Processing

Digital images of serial sections were acquired with a resolution of 1404 × 936 pixels (downsized from the original camera resolution of 5616 × 3744 pixels), which corresponded to 270 × 180 µm when the 50× objective was used. GIMP 2.10 (S. Kimball, P. Mattis and the GIMP Development Team) and ImageJ 2.3.0 (U. S. National Institutes of Health) running on a MacBook laptop computer (Apple Inc., Cupertino, CA, USA) were employed. Identical image-processing procedures were used as described in previous reports [17,18].

### 2.4. Tracing Cell Files Derived from Vascular Initials

The lineages of cells produced by individual initials were traced by examining the images of serial sections and constructing 3D virtual objects using those same serial sections. The plerome, vascular initials, pericycle, LMX daughter cells (the first cells generated from the vascular initials), and the first “collar cell” (established LMXs in *Zea* roots are surrounded by distinct peritracheal parenchyma cells [8]) were marked by colors and investigated. Standard HTML color series (https://www.w3schools.com/colors/colors_names.asp; last accessed on 10 March 2024) were used. The marking of the LMXs was easily performed in serial transverse sections proceeding acropetally if started at the section around 100 μm from the root cap junction (RCJ).

### 2.5. Counting the Numbers of Pericycle Cells, Vascular Cells, and Vascular Initials

The numbers of pericycle cells, vascular initials, and vascular cells (i.e., all the cells internal to the pericycle) were counted using photo images of serial sections from the apex of the vascular cylinder (AVC; demarked by the distal surface of the plerome) to 400 μm from the RCJ. The numbers and average cross-sectional areas of vascular cells were obtained using the “Analyze Particles” tool provided by ImageJ. A transparent layer was laid over the original digital micrograph of each section, then a distinctive dot was placed over each cell observed. The average cross-sectional area of the vascular cylinder within the pericycle was divided by the number of cells to provide the average cell area. The number of vascular initials was counted as the cells internal to the pericycle present in the first layer basipetal to the plerome. The number of cells (pericycle cells or vascular cells) increased with the distance from the RCJ.

## 3. Results

### 3.1. Plerome, Vascular Initials and LMXs of Teosinte

**Figure 2:**
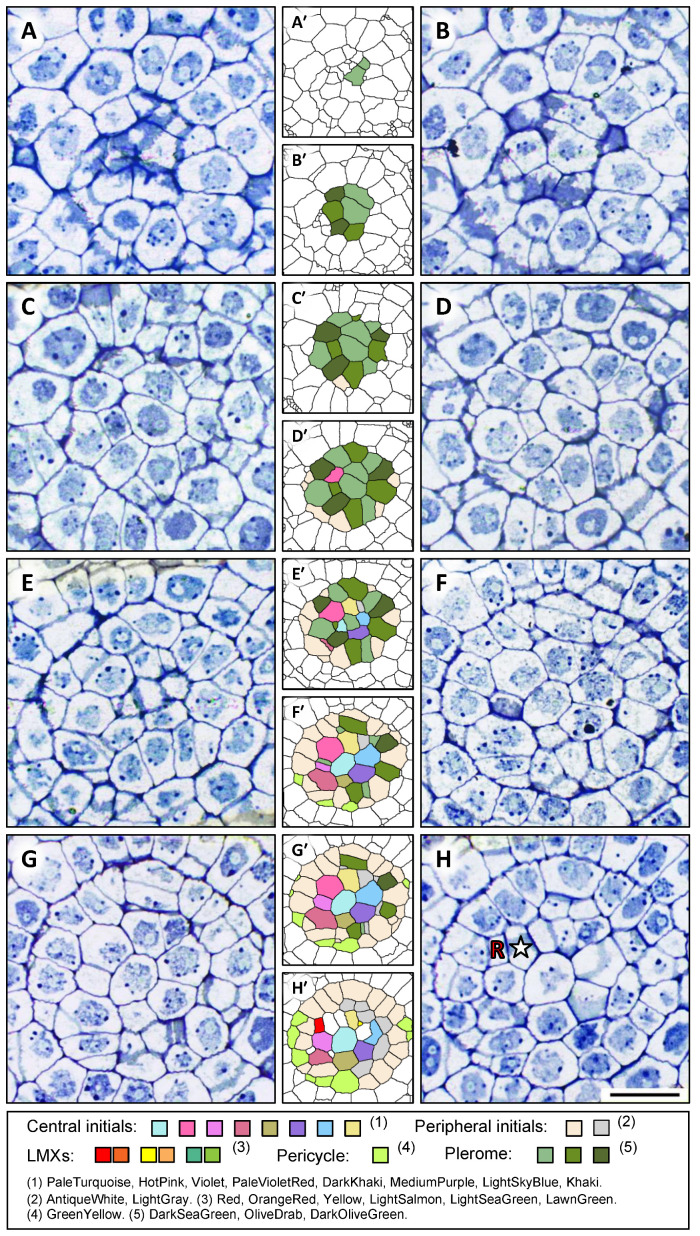

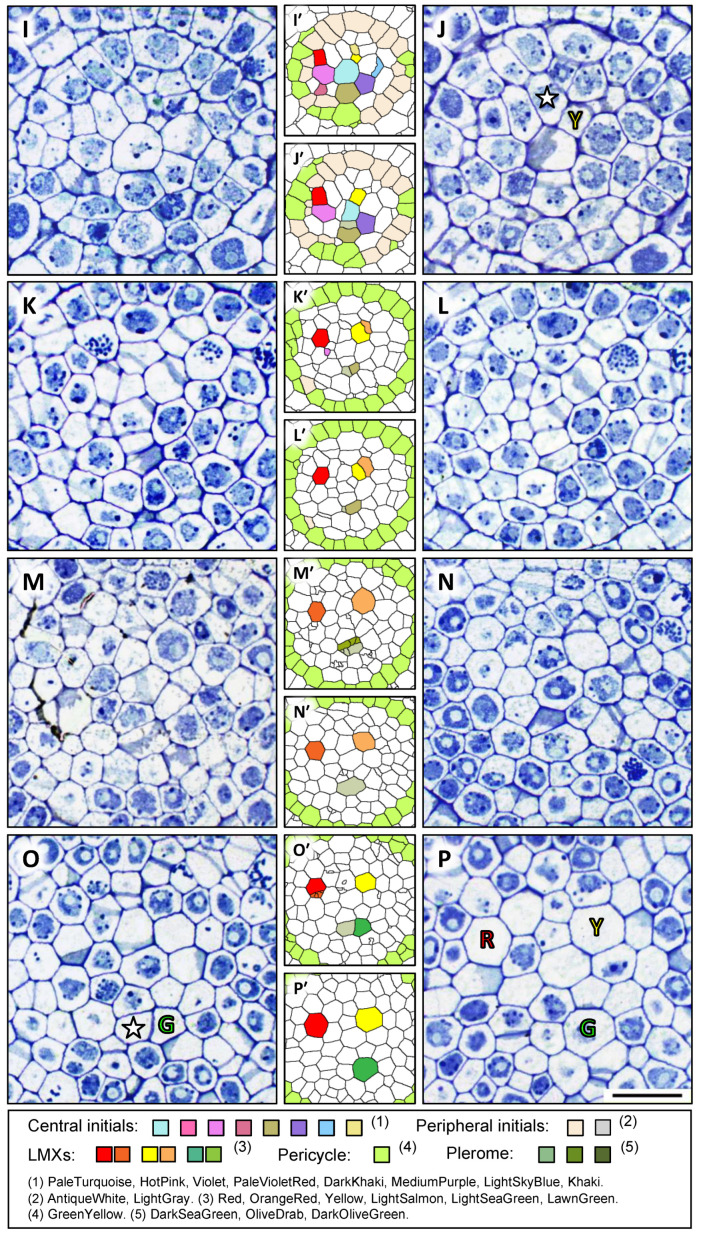
Micrographs with the corresponding processed digital images of selected transverse sections of a root apex of teosinte. Each processed digital image (e.g., **A**′ beside **A**) is marked with several colors for easy tracking of all the cells. (**A**′–**O**′) Different colors are assigned to the plerome cells, central initials, peripheral initials, pericycle, and LMX. Panels (**A**–**D**) show the sections at 25, 26, 30 and 32 μm from the RCJ, respectively, with A being the distal face of the plerome at AVC; (**E**–**H**) show the sections at 34, 37, 39 and 40 μm from the RCJ, respectively. An LMX (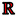) and a collar cell (☆) that differentiated from the initial in (**G**′) (HotPink) can be recognized in (**H′**). Scale bar = 20 µm. Selected transverse sections of a root apex of teosinte from 41 to 80 μm from the RCJ. The sections shown in (**I**–**P**) were 41, 42, 49, 50, 58, 63, 69 and 80 µm from the RCJ, respectively. Pericycle cells (GreenYellow) produced by peripheral initials (AntiqueWhite) can be seen in (**I′**,**J**′). An LMX (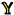) and a collar cell (☆) can be recognized in (**J**). Two LMXs (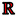 and 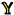) can be seen in (**K**–**L**). The daughter cell derived from a peripheral initial (LightGray) in (**L**′–**O**′) divided to form the third xylem initial (a secondary LMX initial, 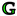) and a collar cell (☆) as shown in (**O**). All three LMXs (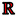, 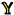 and 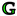) are shown in (**P**). Scale bar = 20 μm. Three LMXs that we labeled red, yellow, and green in (**P′**) were derived from the corresponding original points with the same colors (**H′,J′,O′**), and the progression of the development of the LMXs were observed.

The structures, identities, and spatial distributions of the cells at the center of the root apex of teosinte were analyzed. Figure 2 shows sequential images of transverse sections and the corresponding colorized digital images that allowed us to characterize procambial cells in virtual constructs from a teosinte primary root from the AVC (25 µm from the RCJ) to 80 µm from the RCJ. Figure 2A shows the section image at the AVC in which almost all the cells belong to the dermatogen or periblem, except for a small central area that is the distal limit of the plerome. This small central area becomes a larger group of cells that form the apical domain of the VC (Figure 2B). The group consists of two central cells, with multiple cells surrounding them forming a uniseriate disk (Figure 2C,D), and corresponds to the “plerome” shown in Figure 1. The plerome (colored by three shades of dark green: DarkSeaGreen, OliveDrab, and DarkOliveGreen) comprises the most distal cells of the VC (Figure 2A–D). The color names used here and hereafter were chosen from 140 named colors known as the “HTML Color Names” (standard HTML color series: https://www.w3schools.com/colors/colors_names.asp; last accessed on 10 March 2024).

Vascular initials appear just basipetally to the plerome (Figure 2E) and can be seen to comprise seven cells surrounding a central cell and other cells on the periphery of that central group of nine that together produce the pericycle and the founding cells of the remaining procambial tissue (Figure 2F,G). Consequently, we classified the vascular initials into “central initials” and “peripheral initials”. In the figure, we made these cells distinctive by coloring the center cell with PaleTurquoise, the seven surrounding cells with different colors (HotPink, Violet, PaleVioletRed, DarkKhaki, MediumPurple, LightSkyBlue, and Khaki), and the peripheral cells with AntiqueWhite and LightGray (Figure 2E′–H′). After subsequent development, the pericycle (GreenYellow, Figure 2K′–P′) and three LMXs (Figure 2P,P′) were completely apparent.

By tracing the serial sections starting at around 100 µm from the RCJ and examining the sections in order progressively closer to the plerome, the origins of the LMX files were found easily. Three LMXs that we labeled red, yellow, and green in Figure 2P′, derived from the corresponding original points with the same colors (Figure 2H′,J′,O′), and the progression of the development of the LMXs were observed. The first appearance of an LMX cell produced by a vascular initial was observed in the section 40 μm from the RCJ. A vascular initial cell (HotPink in Figure 2G′) had divided into an LMX (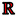) and a collar cell (☆), shown in Figure 2H. The differentiation of the second LMX (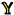) could be seen in the section 42 µm from the RCJ and the corresponding initial cell (Khaki in Figure 2I′) divided into an LMX (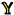) and a collar cell (☆ in Figure 2J). The differentiation of the third LMX (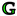) followed a slightly more complicated process. The daughter cell produced by one of the central initials (DarkKhaki, Figure 2I′) underwent a subsequent cell division once at 58 µm from the RCJ in the axial plane (Figure 2M). These derivative cells differentiated into an LMX element (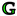) and a collar cell (☆) at the section 69 μm from the RCJ (Figure 2O). All the LMXs (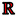, 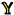, and 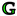) could be detected at 80 µm from the RCJ (Figure 2P,P′).

### 3.2. Cells Detected as Vascular Initials in Teosinte Promeristems

**Figure 3:**
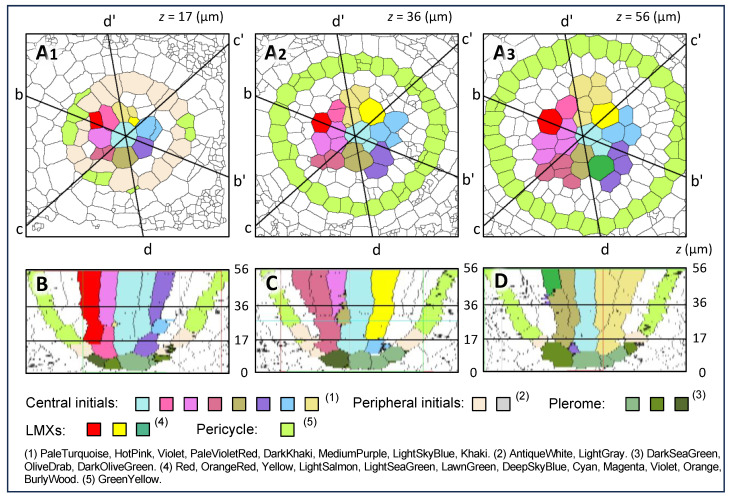
Virtual tracing of cell files derived from the central initials and peripheral initials of a root apex of teosinte. (**A_1_**–**A_3_**) are images constructed from the transverse sections at 17, 36 and 56 µm from the apical end of the VC, respectively. AntiqueWhite-colored cells are peripheral initials. GreenYellow indicates pericycle cells. (**B**–**D**) are the virtual longitudinal sections constructed from serial transverse sections of the root tip by using the plug-in “Volume Viewer” in ImageJ.

The characteristics of the cells located basipetally to the plerome were examined. Figure 3A_1_–A_3_ are images constructed from transverse sections at *z* = 17, 36 and 56 µm from the AVC, respectively. Figure 3B–D show the virtual longitudinal section images of the cutting planes of b–b′, c–c′ and d–d′ in Figure 3A_1_, A_2_, and A_3_, respectively, as constructed with the use of the “Volume Viewer” of ImageJ. In these figures, specific colors were used to trace the ontogenetic lineage of each cell from its central initial, i.e., the lineage of a file of cells originating from a given initial can be visualized by its color labeling.

It was found through the above inspection that all the cells occupying the middle region of the procambium were derived from the central cells seen in Figure 2G–G′, whereas the cells occupying the outer region of the procambium, including the pericycle (GreenYellow), were derived from the peripheral initials (AntiqueWhite and white). The youngest cells of the pericycle sometimes appeared at the position *z* = 17 µm (colored GreenYellow), and they were produced from peripheral initials (Figure 3A_1_).

Our data showed that all the cells of the procambium were derived from these two groups of initial cells (Figure 2G) immediately basipetal to the plerome. Therefore, these cells should be regarded as vascular initials, and they could be classified into central and peripheral initials. Furthermore, it should be mentioned that the color AntiqueWhite, which indicates peripheral initials in the transverse section in Figure 3A_1_, appeared in the reconstructed virtual longitudinal images (Figure 3B–D).

### 3.3. 3D Structure of the Plerome and Vascular Initials of Teosinte

**Figure 4:**
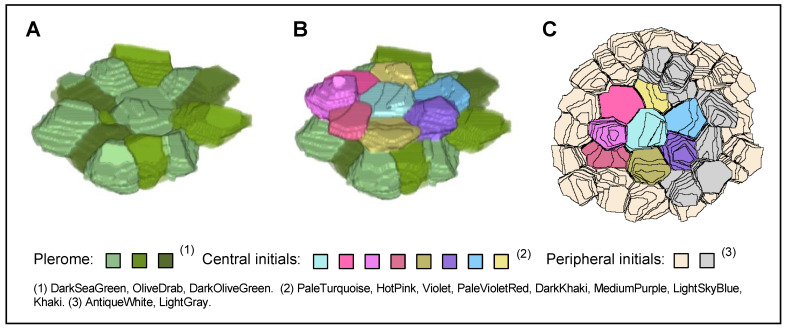
Three-dimensional imaging of the root plerome and vascular initials of teosinte. (**A**) Oblique view of a virtual 3D image of the plerome basipetal face. (**B**) Similar perspective view of a 3D image with central initials added, and (**C**) view of the basipetal face of all the vascular initials, reconstructed from the serial transverse sections of teosinte.

A virtual 3D image of the plerome and vascular initials was constructed from the serial sections shown in Figure 2. Figure 4A shows an oblique “bird’s-eye” view of the basipetal face of the plerome. It consists of two central cells with a ring of cells surrounding them. Figure 4B shows a basipetal perspective view of the layer of central vascular initials immediately basipetal to the plerome that consists of seven cells surrounding a central cell. The entire set of vascular initials, consisting of central and peripheral initials, is shown in a view normal for the plane of the plerome in Figure 4C.

### 3.4. 3D Structure of LMXs of Teosinte

**Figure 5:**
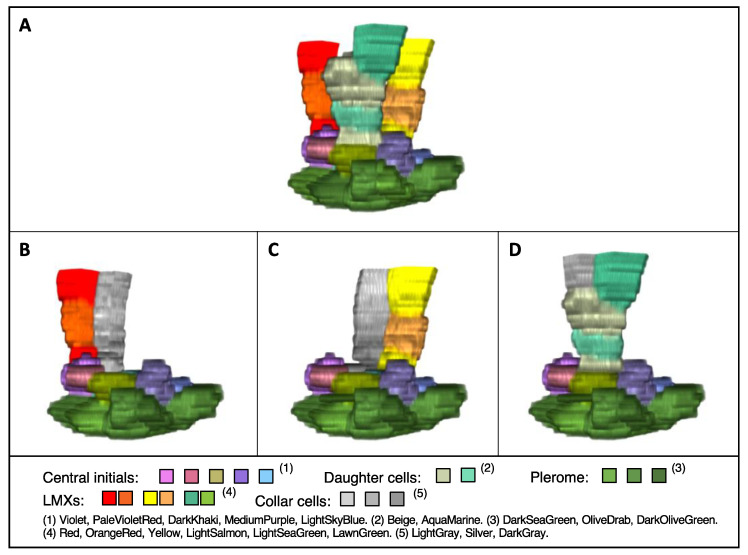
Virtual 3D images of LMXs with central initials and plerome constructed from serial transverse sections of teosinte at 17 to 80 μm from the RCJ. (**A**) Three-dimensional image featuring three LMXs. (**B**–**D**) Three-dimensional images of each LMX and the associated collar cell lineage, each derived and developed from one of the vascular initials. Note: These images were processed using the plugin “3D Viewer”, so the colors in the images are not exactly the same as the colors shown in the color list.

A virtual 3D image comprising LMXs, central initials, and the plerome was constructed (Figure 5A–D). Figure 5A shows the whole image with a vantage similar to that in Figure 4A,B. Figure 5B–D show the 3D images of each individual LMX with its first collar cell, which was produced by the same vascular initial. The LMXs shown in Figure 5B,C were generated directly from the central initials. However, the LMX (and its first collar cell) shown in Figure 5D was derived from a daughter cell divided from a central initial.

### 3.5. Plerome, Vascular Initials, and LMXs of Rice

**Figure 6:**
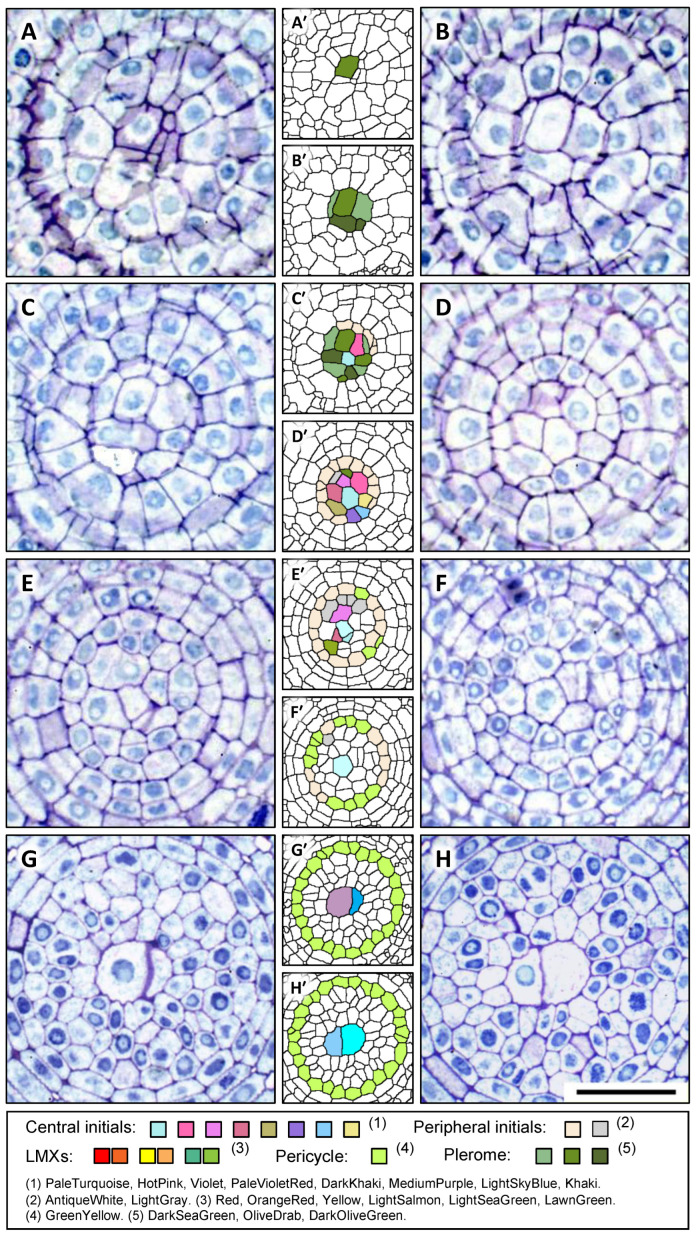
Micrographs with the corresponding processed digital images of selected transverse sections from a root apex of rice. (**A**–**F**) are the sections at 06, 08, 09, 10, 17 and 22 µm from the RCJ, respectively. The figure inserted beside each section (e.g., **A′** beside **A**) is the digitally processed image and marked with identifying colors. Different color groups are given to the plerome, central initials, and peripheral initials (**A′**–**D′**), consistent with Figure 2. (**G**,**H**) are the sections at 43 and 48 μm from the RCJ, respectively. Different color groups are given to the central initials, peripheral initials, and pericycle (**E′**–**H′**), consistent with Figure 2.

Figure 6 shows successive images of serial transverse sections of a primary root of rice at 6 µm (AVC) to 48 μm from the RCJ. Figure 6A shows the image at the AVC, in which almost all the cells belong to the dermatogen–periblem complex, except for a small central area that contains the apical surface walls of the plerome. This small central area adjoins a group of cells that form the apical domain of the VC, the plerome (Figure 6B). The plerome (colored by three shades of dark green: DarkSeaGreen, OliveDrab and DarkOliveGreen) is composed of a number of cells at the apex of the VC (Figure 6B–D) and is a single layer of cells like teosinte, but a center cell is not obvious. Vascular initials were found basipetal to the plerome (Figure 6C′) and formed seven cells surrounding a center cell (the central initials), which was surrounded by other cells, the peripheral initials (Figure 6D′). The peripheral initials colored AntiqueWhite mainly work to generate pericycle cells (GreenYellow).

**Figure 7:**
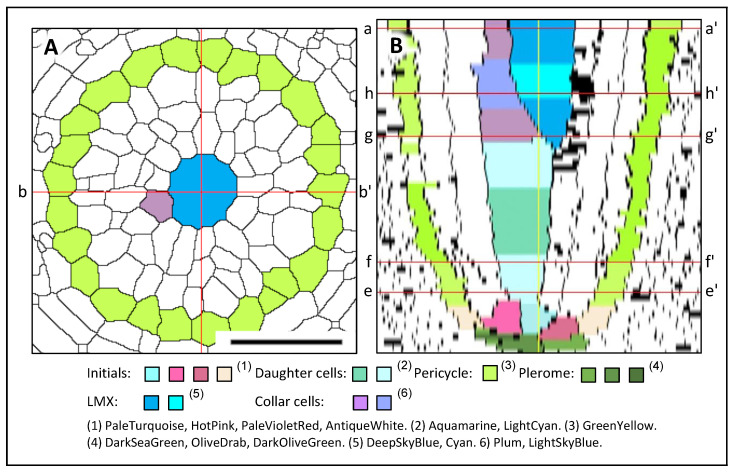
LMX development in a rice primary root. (**A**) Processed digital image of the transverse section at 64 μm from the RCJ. (**B**) Median longitudinal virtual image of the VC constructed from serial transverse sections taken 0–48 µm from the RCJ, section examples shown in Figure 6, with the plerome and initials highlighted at the apex of the VC. A daughter cell (LightCyan), which was generated from the center cell of the central initials, propagated linearly up to about 42 μm from the RCJ. At that position, an LMX initial was obliquely divided from the daughter cell descendant (colored Plum; corresponds with Figure 6G,H) that differentiated as a collar cell (Figure 6B′,C′). The cutting planes e–e’, f–f’, g–g’ and h–h’ in (**B**) correspond to the transverse sections (E′–H’) in Figure 6, respectively. Cutting plane a–a’ corresponds to the transverse section in (**A**). Cutting plane b–b’ corresponds to the longitudinal section shown in (**B**). Scale bar = 50 μm.

Only one LMX is produced by the procambium of a rice primary root. The LMX is generated from a daughter cell derived from the center initial that subsequently divided longitudinally at 43 μm from the RCJ (PaleTurquoise; Figure 6E′). The other cell that divided from the same daughter cell (Figure 6G,H and Figure 7) became the first of the peritracheal “collar” cells that ultimately surrounded the LMX. Figure 7A shows a transverse section of rice at 64 μm from the RCJ and corresponds to the a–a′ cutting plane in the longitudinal image in Figure 7B.

Figure 7B is a virtual longitudinal image constructed from serial transverse sections using the Volume Viewer of ImageJ. The central initials, peripheral initials, pericycle, plerome, daughter cells and the LMXs are shown.

### 3.6. Arrangement of Initials That Generate LMXs

**Figure 8:**
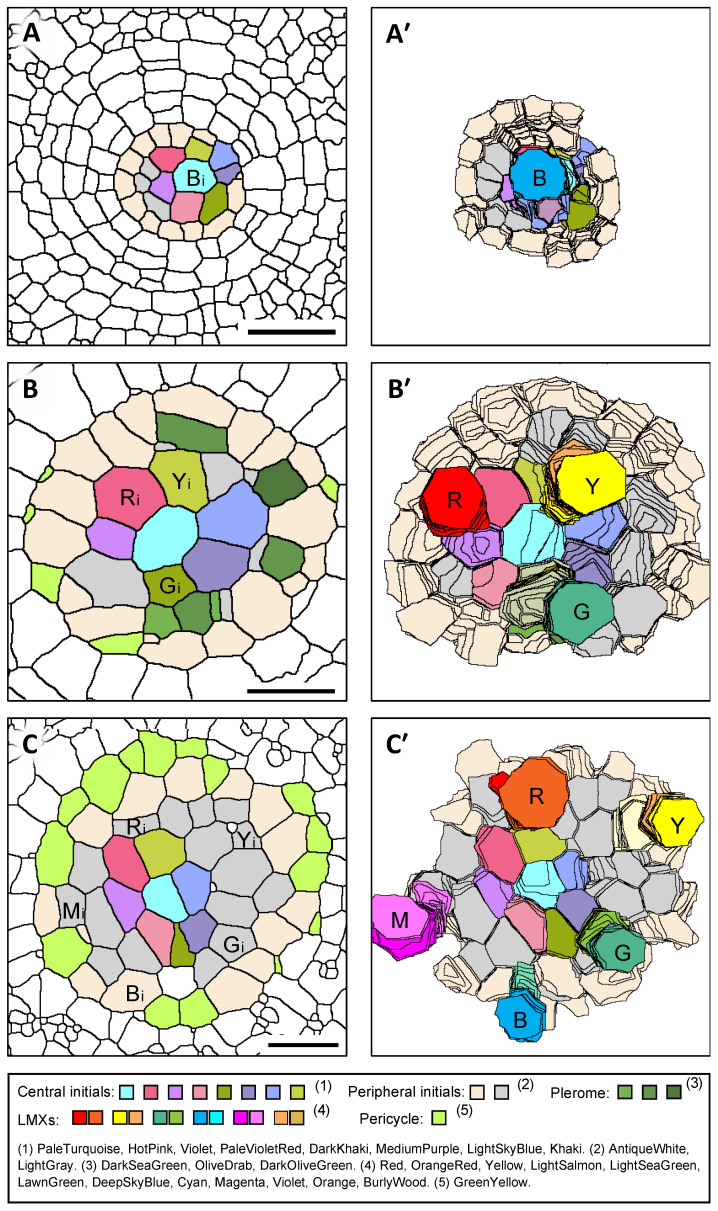
The origin and subsequent early development of the LMXs of the roots of three taxa of Poaceae. (**A**,**A′**) The single LMX of the primary root of rice (**B**) originated from the central initial (Bi). (**B**,**B′**) The multiple LMXs of the primary root of teosinte (R, Y, and G) originated from the central initials Ri, Yi and Gi. (**C**,**C′**) The multiple LMXs of the primary root of a sweet corn primary root (R, Y, G, B, and M) originated from the peripheral initials Ri, Yi, Gi, Bi and Mi. Scale bar = 20 µm.

From the close observations of the LMX initiations in the 3D reconstructions, it was found that the number of LMXs and the locations of their initial cells depended on the taxon and category of the roots. Figure 8 shows examples of the arrangements of LMXs and the original cells from which the LMXs were generated in the primary root procambium. The left side of Figure 8A–C shows the transverse sectional views of the vascular initials layer, showing the positions of origin of the LMXs, and the right side (A′, B′, C′) shows the views of the basipetal faces of the vascular initials layer, showing each LMX’s distal (youngest) cells, the central initials, and peripheral initials in the 3D reconstructions. For example, an LMX and its initial are marked in blue shades as “B” and “Bi”, respectively. The primary roots of barley and rice, whose LMXs derive from the center cell of the central initials, are classified as type A–A′. The primary roots of teosinte and the nodal roots of barley and rice, whose LMXs derive from the central initials, are classified as type B–B′. The nodal roots of teosinte and corn, whose LMXs derive from the peripheral initials, are classified as type C–C′. 

### 3.7. Plerome, Vascular Initials, and LMXs of Sweet Corn and Barley

**Figure 9:**
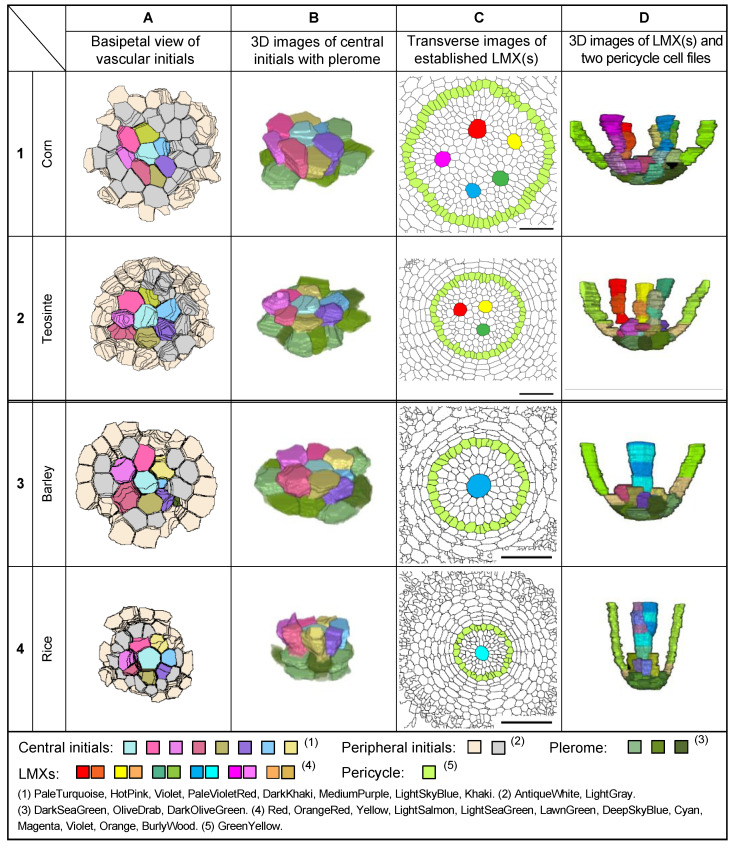
Characteristics of the primary root tip procambia of four taxa (1–4) in the Poaceae as seen in enhanced section images and 3D constructions. (**A**) Views of the basipetal face of the vascular initials layers, (**B**) oblique perspective of 3D constructions of the central initials and plerome cells (shades of green), (**C**) transverse images of the VC at 100 µm from the RCJ, and (**D**) 3D images from the primary root tips of the LMX(s) and two pericycle cell files associated with their plerome and vascular initials layer. Scale bar = 50 µm.

**Figure 10:**
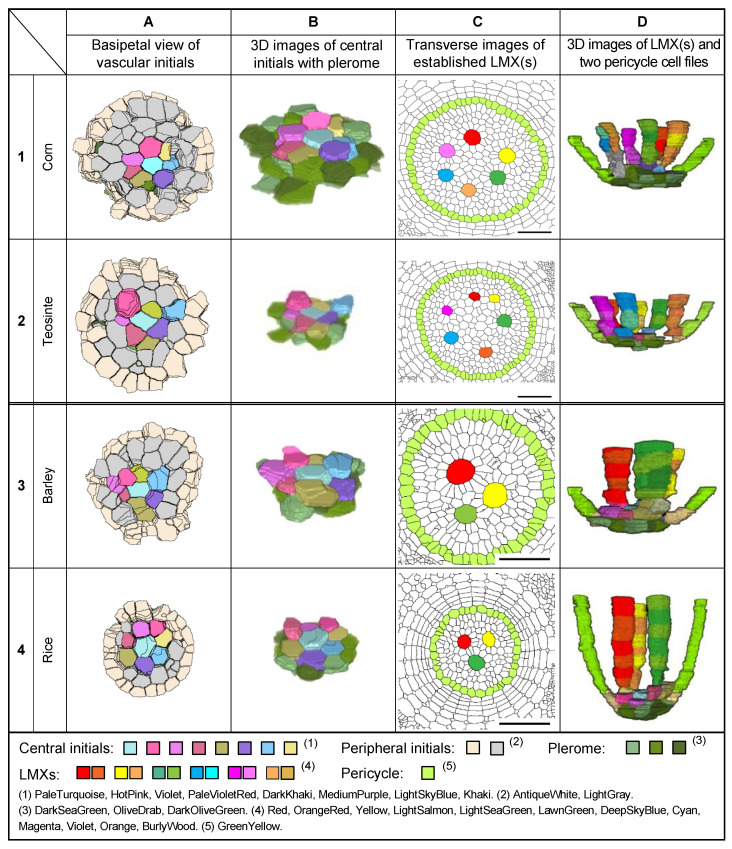
Characteristics of the nodal root tip procambia of four taxa (1–4) in the Poaceae as seen in enhanced section micrographs and 3D constructions. (**A**) Views of the basipetal face of the vascular initials layers, (**B**) 3D images of oblique basipetal views of the central initials with the plerome, (**C**) transverse images of the VC at 100 µm from the RCJ, and (**D**) 3D images of the LMX(s) of the nodal roots of four kinds of Poaceae. Scale bar = 50 µm.

**Figure 11:**
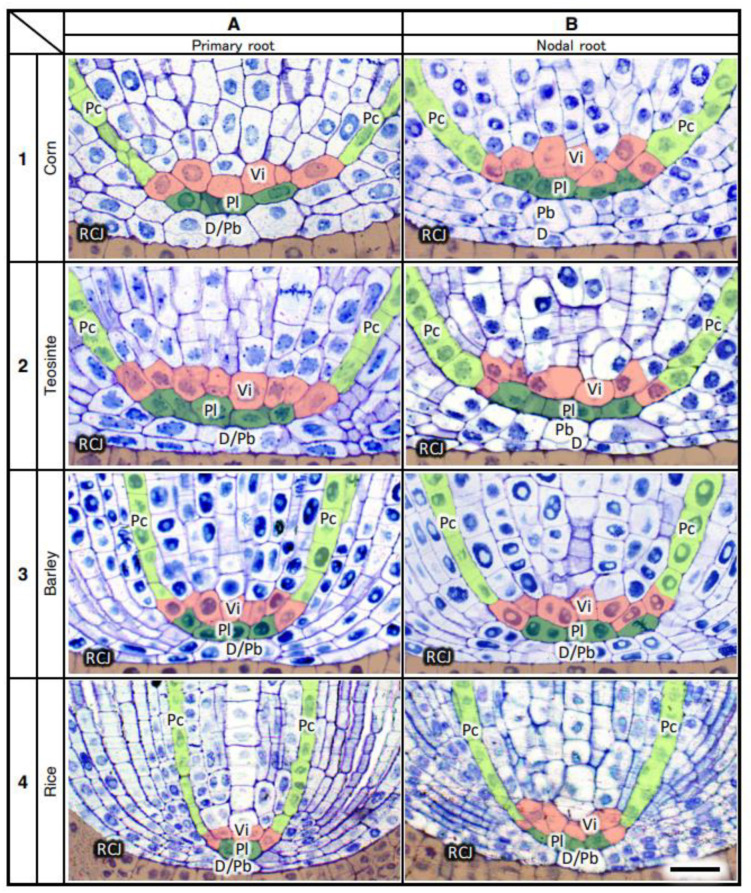
Longitudinal section images of the root apex of the primary and nodal roots (**A** and **B**, respectively) of four taxa (1–4) in the Poaceae. The thickness of the sections was 2 μm. D, dermatogen; D/Pb, dermatogen–periblem complex; Pb, periblem; Pc, pericycle; Pl, plerome; RCJ, root cap junction; Vi, vascular initial. Bar = 20 µm.

The structures of the plerome and vascular initials in the root apices of sweet corn and barley were in accord with those of teosinte and rice (Figure 9, Figure 10 and Figure 11). In the primary root apex of corn with five LMXs, the first signs of differentiation of an LMX occurred at 35 μm from the RCJ. The differentiation of the second, third, fourth and fifth LMXs occurred at 39, 40, 40 and 41 μm from the RCJ, respectively.

In the case of the promeristem of the primary root of barley, a single LMX began differentiation at 38 μm from the RCJ and was derived from the center cell of the central initials, as in rice. For the nodal roots of barley with three LMXs, their differentiation began at 40, 41 and 44 μm from the RCJ, respectively.

### 3.8. Structure of the Root Promeristems of Four Taxa in the Poaceae 

Figure 9 shows examples of (A) the basipetal face-view images of the vascular initials, (B) virtual 3D images of the central initials and their associated pleromes, (C) transverse images of fully differentiated VCs emphasizing the locations of LMXs and pericycle cells, and (D) virtual 3D images of the LMX origins for the primary roots of four types of Poaceae seedlings. Figure 10 shows the same categories as Figure 9, but for nodal roots. The primary and nodal roots of these poacean seedlings shared similar procambium cell-level structural patterns. In these images, the plerome and vascular initials are marked with distinctive colors. The plerome was organized transversely in a uniseriate layer, while the vascular initial cells were located immediately basipetal to the plerome (Figure 11).

In the primary roots of Poaceae, the dermatogen and periblem (located acropetally from the apex of their VCs) formed a single layer as a complex, whereas for the nodal roots of corn and teosinte, their dermatogens and periblems were separated into different layers. The promeristems of the nodal roots of barley and rice were noticeably broader than their corresponding primary roots (Figure 11).

### 3.9. Increase in Pericycle Cell and Vascular Cell Numbers

**Figure 12:**
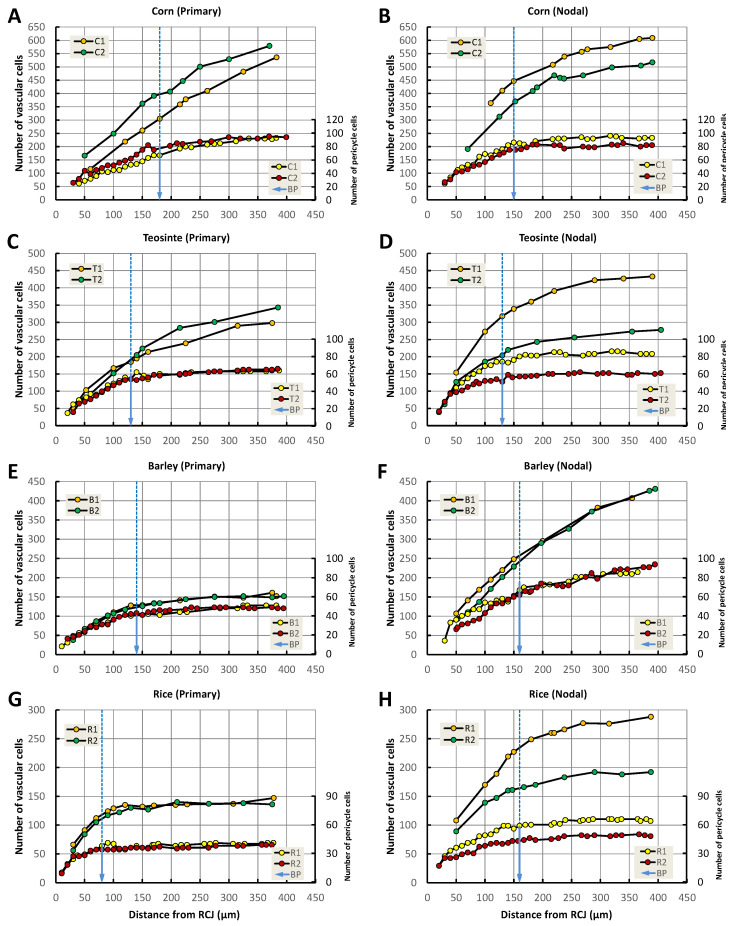
Numbers of pericycle cells and vascular cells of two typical primary and two nodal roots of four poaceans at specified distances from the RCJ. (**A**,**B**) corn, (**C**,**D**) teosinte, (**E**,**F**) barley, (**G**,**H**) rice. Blue arrow: the boundary point (BP) defined as the locations where the rapid rate of increase in the pericycle cell number declined notably and began to trend toward zero change.

The transverse numbers of pericycle cells and vascular cells in roots of the four seedling taxa were determined at specified distances from the RCJ (Figure 12). Two phases in the pattern of the radial cell proliferation were perceived: an early period when the number of cells in sequential transverse planes of a VC rapidly increased and a late period when the rate of increase was more gradual. We defined a boundary point (BP) along the VC axis where the rate of increase of the cell number shifted from a rapid rate to a slower, more gradual change. The BP is indicated for two typical roots in Figure 12, with a blue arrow for the pericycle cells in each figure panel. In almost all the roots, the pericycle cells stopped increasing in number beyond the BP, and the vascular cells continued to increase in number only slightly or more gradually than before the BP.

**Figure 13:**
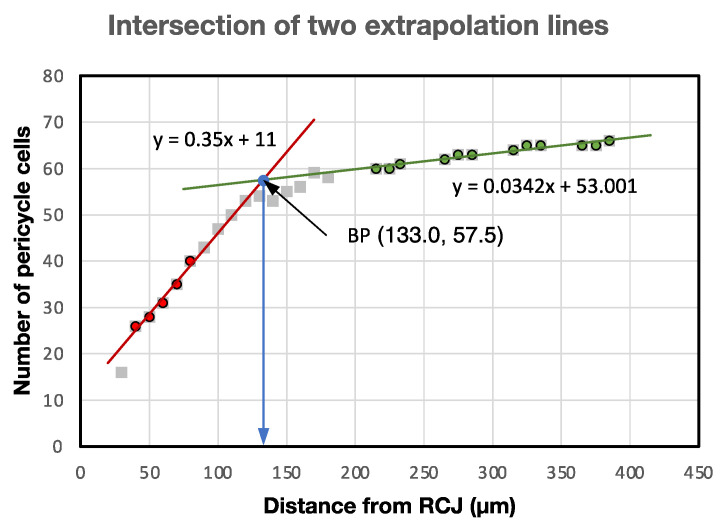
Explanatory diagram typical for finding the boundary point (BP) at the intersection of a forward extrapolation line (red dots) and backward extrapolation line (green dots) taken from teosinte primary root pericycle cell number data measured in the young, rapidly proliferating part of the VC tip and the older, more gradually expanding part of the VC, respectively. Gray squares without colored dots are the measured transitional data between the two zones. Red dots: lower part (40–80 µm from RCJ) of the measured data used for forward extrapolation. Green dots: higher part (200–400 µm from RCJ) of the measured data used for backward extrapolation. BP: boundary point.

Figure 13 shows an explanatory diagram typical for finding the boundary point (BP) using the measured data from a teosinte primary root, where the *x*- and *y*-axes are the distance from the RCJ and the number of pericycle cells, respectively. The BP was located mathematically at the intersection of two extrapolation lines. One was given as a forward extrapolation line drawn toward the root base from the acropetal, rapidly expanding part (40–80 µm from RCJ; red dots) of the procambium. The other was given as a backward extrapolation line drawn toward the root tip from the gradually expanding basipetal part (200–400 µm from RCJ; green dots) of the procambium. The transitional data (gray squares) cross the BP (Figure 13).

### 3.10. Number of Vascular Cells vs. Number of Vascular Initials

**Figure 14:**
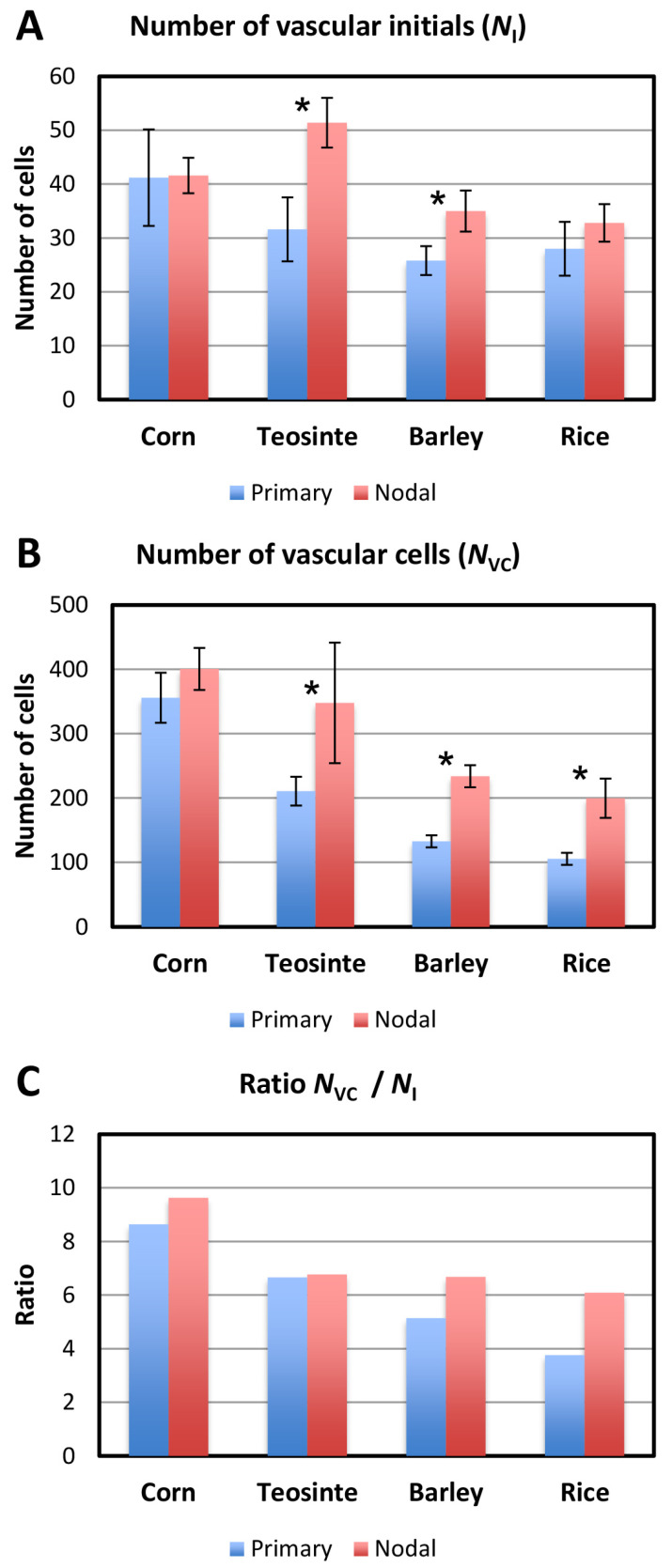
Numerical analysis of the relationship between the vascular initials and the number of vascular cells in the transverse plane through the vascular cylinder (VC) at the calculated boundary point (BP). (**A**) Number of vascular initials, (**B**) number of vascular cells, and (**C**) the ratio of the mean number of vascular cells (*N*_vc_) to the mean number of vascular initials (*N*_i_) for the primary and nodal roots of the four taxa (corn, teosinte, barley, and rice). * *t*-test, different at *p* < 0.05; error bar = standard deviation; *n* = 5 each.

The number of vascular initials of the primary and nodal roots of each species was counted in the vascular initials layer as described above. We regarded all the cells in the sections 8–11 µm from the AVC as vascular initials. The term “vascular cells” was defined as all the cells confined by and including the pericycle. Each value shown in Figure 14 is the average of five specimens for each taxon (corn, teosinte, barley, and rice). The numbers of vascular initials (Figure 14A) showed slight differences among the taxa. The numbers of vascular cells at the BP showed considerable differences among the taxa and root categories, except for corn, whose primary and nodal roots did not differ in terms of these parameters (Figure 14B). Figure 14C shows the ratio of the number of vascular cells (*N*_vc_) to the number of vascular initials (*N*_i_) for the primary and nodal roots of the four taxa. In the figure, we report the ratio *N*_vc_/*N*_i_ by taxon in decreasing order.

### 3.11. Diameter of the VC, Average Size of Vascular Cells, and the Number of LMXs

**Figure 15:**
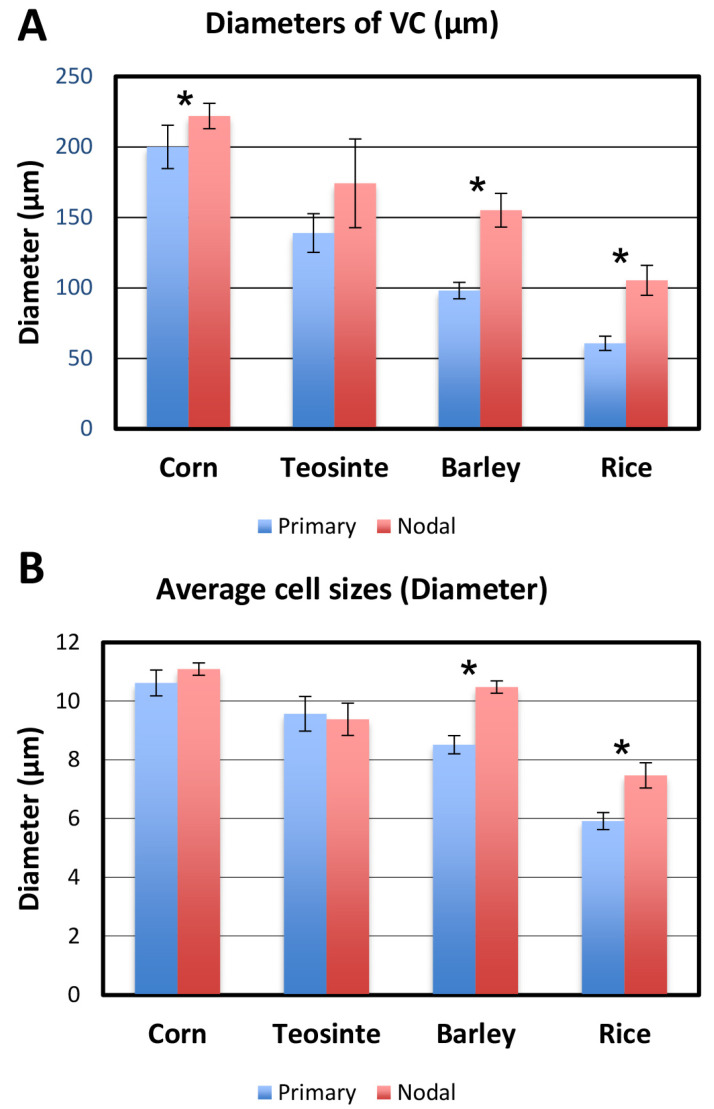
Numerical comparisons of the vascular cylinders (VCs) and cells confined therein at the BP for the primary and nodal roots of the four taxa (corn, teosinte, barley, and rice). (**A**) Average diameters of the VCs and (**B**) average diameters of individual vascular cells. * *t*-test, different at *p* < 0.05; error bar = standard deviation; *n* = 5 each.

Figure 15A shows the diameters of the primary and nodal root procambia of the four taxa at the BP. Except for corn, it was found that the diameters of the primary roots were generally smaller than those of the nodal roots within each taxon and that there was no significant difference in the diameter at the BP among the nodal roots of corn, teosinte, and barley. The rice roots, primary and nodal, were smaller than those of the other taxa tested. The average size (diameter) of the vascular cells in the primary and nodal roots of corn, teosinte and barley was similar, whereas that of rice was clearly smaller than the others (Figure 15B).

**Figure 16:**
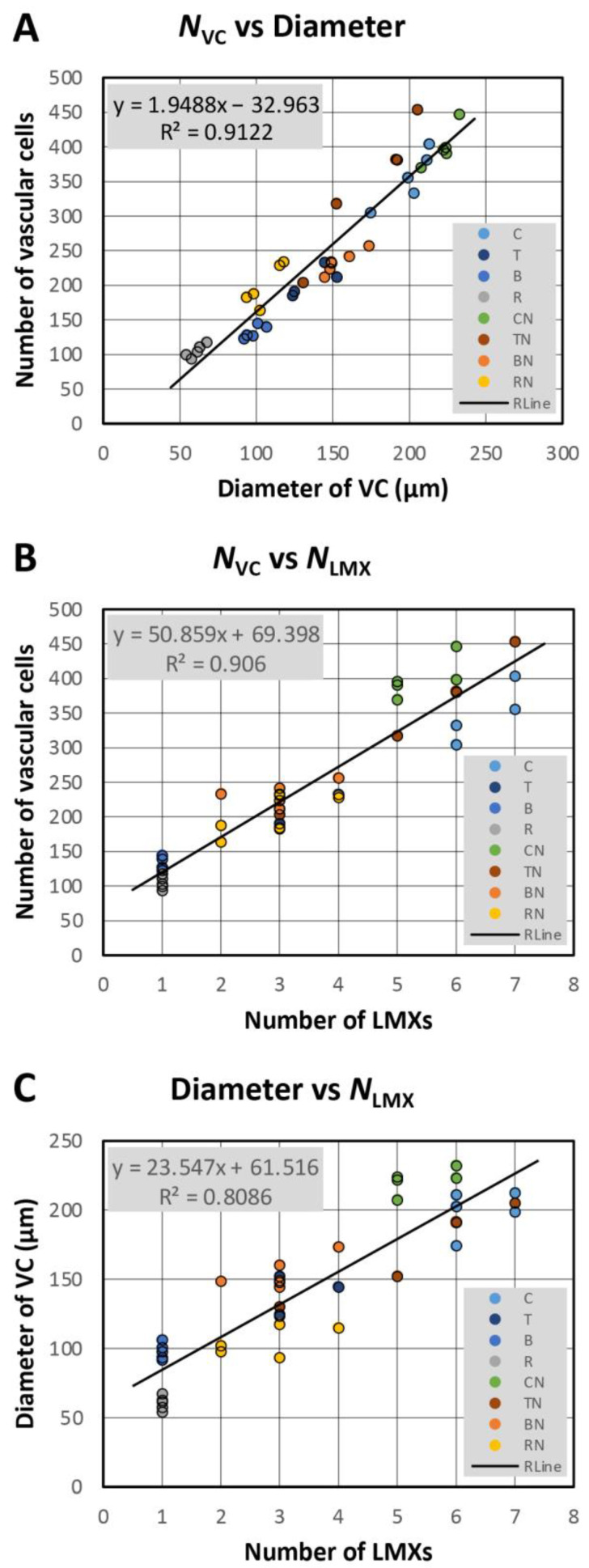
Numerical analysis of three parameter relationships for the individual primary and nodal roots of the four taxa (corn, teosinte, barley, and rice). (**A**) The relationships between the number of vascular cells (*N*_vc_) and the diameter of the VC, (**B**) the number of vascular cells (*N*_vc_) and the number of LMX cell files, and (**C**) VC diameter and the number of LMX cell files (*N*_LMX_). Primary roots: C, corn; T, teosinte; B, barley; R, rice. Nodal roots: CN, corn; TN, teosinte; BN, barley; RN, rice. Rline: the solid line in each graph is the regression line calculated from the measured data.

Figure 16A shows the relationship between the number of vascular cells (*N*_vc_) and the diameter of the VC at the BP for all the data (five samples for each root type of the four taxa). The solid line in Figure 16A is the regression line calculated from the measured data. A strong correlation (*r*^2^ = 0.9122) is found between the number of vascular cells (*N*_vc_) and the diameter of the VC.

Figure 16B shows the relationship between the number of vascular cells (*N*_vc_) and the number of LMXs (*N*_LMX_) at the BP for all the data. The solid line in Figure 16B is the regression line calculated from the measured data. A strong correlation (*r*^2^ = 0.906) is found between the number of vascular cells (*N*_vc_) and the number of LMXs (*N*_LMX_).

Figure 16C shows the relationship between the diameter of the VC and the number of LMXs (*N*_LMX_) at the BP for all the data. The solid line in Figure 16C is the regression line calculated from the measured data. The correlation (*r*^2^ = 0.8086) between the diameter of the vascular cells (*N*_vc_) and the number of LMXs (*N*_LMX_) is found to be weaker than that given for the number of vascular cells and number of LMXs (Figure 16B).

## 4. Discussion

### 4.1. Description of the Root VC Apical Structure of Four Taxa in the Poaceae

By using recently developed 3D imaging techniques [17,18], the precise structure of the apical region of the VCs in roots was revealed in four poaceans: corn (*Zea mays*), teosinte (*Zea mays* ssp. *mexicana*), barley (*Hordeum vulgare*), and rice (*Oryza sativa*). The plerome histogen occupies the apex of these VCs, and it consists of varying numbers of central cells and peripheral cells, depending on the taxon, in a single layer. As reported previously [8], vascular initials are located immediately basipetally to the plerome, and, by virtue of newly developed methods [17,18], we reassessed the conclusion of Saito et al. [8] that there are multiple layers of initials. The present results suggest the vascular initials layer is uniseriate. The vascular initials layer can be classified into central initials and peripheral initials. Central initials primarily contribute to the generation of cells in the central region of the VC, including LMXs and their parenchymatous peritracheal “collar” cells. Peripheral initials contribute to the generation of the pericycle at its outermost periphery, and internally to some LMXs (depending on the root type), and ultimately, to other vascular cell types, such as proto- and metaphloem sieve-tube members and nondescript parenchymatous cells that are not located in the central regions of the VC (Figure 3). These latter cell types remain targets to be fully assessed using the present methods.

Our current investigation suggests that the pericycle is generated from peripheral vascular initials rather than from the plerome margin, as previously proposed [8]. After germination of the seed, the plerome and vascular initials layer in young seedling root tips appear to be independent of each other, as we observed no sign of the continuity of the cell supply between them in normal growth (Figure 11). According to the classical histogen theory, the plerome is the histogen that generates the cells of the VC tissues [1]. Heimsch [11], in his study of *Hordeum sativum* (barley), used the term “stelar initials” to describe a single layer of cells that are continuous with the pericycle. Clowes [9] used the term “promeristem” in grasses to include initial (cycling) cytogenerative cells and their most recent derivatives in an apical meristem (as do we). Kawata et al. [13,14], after observing the nodal root apex of rice, described the outermost cells in the apical layers of the VCs of rice as “stele initials” and the cells in the center of a VC as “large cells”. Morita and Nemoto [22] reported the stele initials as being located in the plerome; however, they did not mention the relationship between the “large cells” and vascular initials in rice as per Kawata et al. [13,14]. Our goal was to clarify and unify the concepts of functional root apical organization in the closed organizational systems of these important cereal taxa.

We confirmed that the most distal cells of a VC formed the plerome in these taxa, and the large cells located basipetally to that layer are vascular initials, as Saito et al. [8] observed. It was reported previously that the center of the plerome is generally inactive except for rare periodic pulses of mitotic activity anticlinal to the plane of the plerome that produced cells radially. We interpreted such activity as contributing to the production of the pericycle [8]. In the present study, we saw no evidence that the plerome produced cells that would add to the length of the VC. On the basis of the pattern of lengthening of the LMX files, we interpret that it is the vascular initials layer present basipetally to the plerome that constitutes the set of “primordial” cells that is ultimately responsible for the production of the VC, including the pericycle, in all four taxa. This claim was further strengthened by the presence of cells (labeled AntiqueWhite) between the plerome and pericycle (Figure 3, Figure 7B, Figure 9 and Figure 10).

The meaning of “histogen” (a specific “tissue producer”) has shifted from Hanstein’s original conception [1] because more recent evaluations, such as those of Clowes [3,5,9], von Guttenberg [6], and Jiang and Feldman [23], have expanded interpretations of the RAM structure. We use the term here anatomically to refer to the distinct single layers of cells dependably positioned in the promeristem of seedling roots with closed apical organization, and we focus on the layer called the “plerome” [6,7,8] located in the most distal position of the procambium. Our interpretation of the data is that, for the taxa we evaluated, the cell proliferation functions of the plerome may be restricted to the stages of embryogenesis [24,25], and just before entering dormancy, the embryonic plerome divides into a layer that later becomes active vascular initials and a largely quiescent plerome, consistent with Clowes’ “quiescent centre” analysis [5].

Barlow [26] argued that mechanical forces imposed by the surrounding growing tissues are involved in the formation of the quiescent center. Jiang and Feldman claimed that the formation of a quiescent center is required for normal RAM development and function via auxin fluxes and establishment of an “auxin maximum” [23]. Our data are consistent with the plerome being central to quiescent center formation and function.

### 4.2. LMXs Generated from Vascular Initials

The single LMX found in the primary root of rice or barley is produced by the center cell of the central initials, while the multiple LMXs found in the other roots are derived from the central initials (excluding the center cell) or the peripheral initials, depending on the number of LMXs (Figure 8). In the present study, the number of LMXs in the procambium initiated within 100 µm of the RCJ varied depending on the species and root category. Furthermore, when there are more than one, the LMXs are approximately evenly spaced, and each LMX is eventually encircled by peritracheal “collar cells” [8]. We thought that the number and distribution of the LMXs might be under the control of the central initials. Moreover, there might exist a hierarchy between the center cell and the surrounding cells, and the center cell might be playing the role of a control center for the differentiation of LMXs when there is more than one. This is consistent with previous reports of the roles of certain phytohormones, especially auxin [10,23,27,28,29,30].

### 4.3. Boundary Point for the Increase in Pericycle Cell and Vascular Cell Numbers

Kawata et al. [12,14] examined the number of pericycle cells, LMXs, and the diameter of the VC of rice nodal roots, and they reported that the number of pericycle cells and the diameter of the VC gradually increased with distance from the RCJ, but the number of pericycle cells became constant at around 150 μm from the RCJ. Furthermore, there was a correlation between the number of LMXs and the diameter of the VC.

In the present study, we showed that the pericycle cells and vascular cells increased in number with the distance from the RCJ in a coordinated manner (Figure 12), which means the cells did not simply become larger. In addition, it became clear that there are “boundary points” (BPs) along the root axis where the rate of increase of the cell number begins to decline for the pericycle cells and vascular cells (Figure 12, blue arrow).

The concept of a “transition zone” with regard to a growth process that shifts the morphology of root tip growth was proposed by Baluska et al. [31], and that process was clearly connected to causative underlying changes in the cell ultrastructure that shifted the direction of growth from predominantly isodiametric expansion to mainly axially oriented growth. The BP transition we describe is also likely driven by changing underlying physiological conditions in the cells of the promeristem VC with time. The underlying physiological causation is most probably changes in the transport profile and the tissue-level gradients of hormones such as auxin in the promeristem region, as initially proposed by Torrey [27], reviewed by Torrey et al. [28] and Jiang and Feldman [23], evaluated in *Arabidopsis* [10,32,33], and later demonstrated by Mironova et al. [30].

The pericycle is the outermost tissue structure of a VC, and it could be proposed that the steady proliferation of pericycle cells regulates or simply influences the proliferation and differentiation of cells in the procambium, but that assertion could also be circumstantial. The present work showed that, in primary roots of teosinte, LMX vessels were established 80 μm from the RCJ (Figure 2), meaning that the identity of the principal xylem-conducting tissues of the VCs seems to have been established by the level of the BP (Figure 9C and Figure 10C). However, one or two additional LMXs were initiated and developed in the more mature regions basipetal to the BP of corn’s primary roots and barley’s nodal roots. When we examined the structures of several kinds of root tips and compared them to each other, a transverse section of the specimen 100 µm from the RCJ revealed the fundamental structural properties of that specimen with respect to LMX establishment (Figure 9 and Figure 10).

With respect to the primary roots, among the taxa there were significant differences in the pericycle and vascular cell proliferation patterns such that the BPs differed in location, but there was less variation among the nodal roots in that regard (Figure 12). Kawata et al. [12] examined the nodal roots generated from the upper subterranean nodes and the nodal roots generated closer to the scutellar node of rice, and they reported that there were differences in their RAM structures. Therefore, it is difficult to generalize even for nodal roots, given their positional variability. We speculate that nodal roots generated from locations higher on a culm may be more strongly affected by the stem and leaf signals, whereas those generated from lower positions on stems are more strongly affected by the scutellum and growing primary roots. Alternatively, one can speculate that external factors, such as mechanical stressors, may influence the RAM anatomy, though the distances separating the positions of the nodal roots in particular are not large.

### 4.4. Number of Vascular Initials vs. Vascular Cells, Cell Size and LMX

The number of vascular initials and vascular cells in the primary and nodal roots at the BP are shown in Figure 14A and Figure 14B, respectively. The number of vascular cells in the nodal roots at the BP was higher than in the primary roots (Figure 14B). The ratio *N*_vc_/*N*_i_ (Figure 14C) compares the number of vascular tissue cells to the number vascular initials and offers a sense of how prolific the initials were on average. The ratio’s magnitude tended to correspond to the size of the plerome in a given taxon (Figure 11). Corn and teosinte roots had multiple LMXs, had differences in the VC diameters, and differences in the cell size, regardless whether the primary or nodal roots were considered (Figure 15). On the other hand, differences in the diameter and cell size of the VCs were observed between the primary root samples with a single LMX and the nodal roots with multiple LMXs of barley and rice (Figure 15A,B). Kawata et al. [14] reported that there was a correlation between the diameter of the VC and the number of LMXs. We obtained similar results by quantitative analysis (Figure 16C). Furthermore, we obtained a stronger positive relationship between the number of vascular cells and the number of LMXs (Figure 16B).

**Figure 17:**
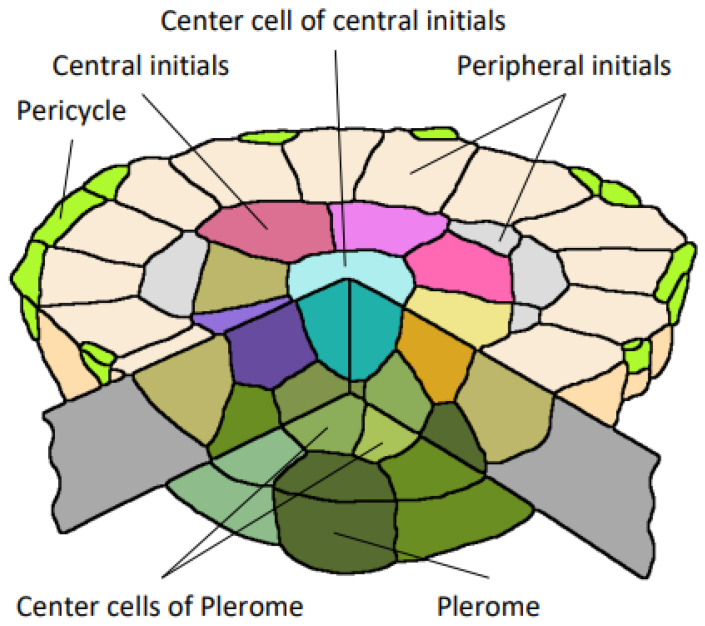
Conceptual diagram of the minimum vascular cylinder apex (plerome and vascular initials).

A diagrammatic conception of the structure of the primary root apex of teosinte based on our observations is presented in Figure 17. With minor adjustments, this concept applies generally to all the root types of the four taxa we examined. We concluded that almost all the LMX cell files originate from initials included in the vascular initials layer basipetally adjacent to the plerome. We intend to extend the use of our new analytical approach to meta- and protoxylem tracheary files, to meta- and protophloem sieve tubes, and to parenchymatous tissues of the vascular cylinders in these four Poaceae taxa in ongoing studies.

## 5. Conclusions

The structure of the root procambia of four taxa of the Poaceae was precisely investigated with respect to the development of the pericycle and LMX vessels and the structure of the plerome and vascular initials layer. Using a new technique [17,18], several structural patterns and histogenic activities relevant to the vascular cylinder (VC) of the promeristem were confirmed. The plerome occupies the apex of the VC, and a layer of vascular initials is located just basipetal to the plerome. These two tissue layers appear to be independent of each other. Once the anatomical pattern is established, the plerome is normally “quiescent” and does not contribute materially to the generation of cells for the VC. Vascular initials can be classified into central initials and peripheral initials and are primordial for all the cells in the VC. The numerical analysis of the VC revealed that (1) several important root structures were established by the ”boundary point” (BP), a location characterized by a shift in the kinetics of cell proliferation, (2) the number of LMXs initiated within 100 µm of the RCJ depended on the species and category of root (primary or nodal), and (3) the diameters of the VCs, number of LMXs, and number of the vascular cells at the BP were strongly related to each other.

## Data Availability

The raw data supporting the conclusions of this article will be made available by the authors on request.
